# Identification of α-Glucosidase Inhibitors from Leaf Extract of Pepper (*Capsicum* spp.) through Metabolomic Analysis

**DOI:** 10.3390/metabo11100649

**Published:** 2021-09-22

**Authors:** Samuel Tilahun Assefa, Eun-Young Yang, Gelila Asamenew, Heon-Woong Kim, Myeong-Cheoul Cho, Jundae Lee

**Affiliations:** 1 National Institute of Horticultural and Herbal Science (NIHHS), Rural Development Administration (RDA), Wanju-gun, Jeonju-si 55365, Korea; sumalew70@gmail.com (S.T.A.); chomc@korea.kr (M.-C.C.); 2 Department of Horticulture, College of Agriculture and Life Sciences, Jeonbuk National University, Jeonju-si 54896, Korea; 3 National Institute of Agricultural Sciences, Rural Development Administration, Wanju-gun, Jeonju-si 55365, Korea; gelilasamenew@gmail.com (G.A.); ksharrier@korea.kr (H.-W.K.)

**Keywords:** capsicum, flavones, polyamines, metabolomics, α-glucosidase inhibition, pepper leaves extract

## Abstract

Metabolomics and in vitro α-glucosidase inhibitory (AGI) activities of pepper leaves were used to identify bioactive compounds and select genotypes for the management of type 2 diabetes mellitus (T2DM). Targeted metabolite analysis using UPLC-DAD-QToF-MS was employed and identified compounds that belong to flavone and hydroxycinnamic acid derivatives from extracts of pepper leaves. A total of 21 metabolites were detected from 155 samples and identified based on MS fragmentations, retention time, UV absorbance, and previous reports. Apigenin-*O*-(malonyl) hexoside, luteolin-*O*-(malonyl) hexoside, and chrysoeriol-*O*-(malonyl) hexoside were identified for the first time from pepper leaves. Pepper genotypes showed a huge variation in their inhibitory activity against α-glucosidase enzyme(AGE) ranging from 17% to 79%. Genotype GP38 with inhibitory activity of 79% was found to be more potent than the positive control acarbose (70.8%.). Orthogonal partial least square (OPLS) analyses were conducted for the prediction of the AGI activities of pepper leaves based on their metabolite composition. Compounds that contributed the most to the bioactivity prediction model (VIP >1.5), showed a strong inhibitory potency. Caffeoyl-putrescine was found to show a stronger inhibitory potency (IC_50_ = 145 µM) compared to acarbose (IC_50_ = 197 µM). The chemometric procedure combined with high-throughput AGI screening was effective in selecting polyphenols of pepper leaf for T2DM management.

## 1. Introduction

Diabetes mellitus is a chronic disease characterized by hyperglycemia. Type 2 diabetes mellitus (T2DM) is the most common form of diabetes that results from genetic susceptibility, environmental factors, and living style. T2DM develops due to insulin resistance and continued damage of pancreatic β-cells which result in increased glucose levels in the bloodstream. Hyperglycemia or high blood glucose level is associated with various complications that could lead to organ failures and death [1,2]. Diabetes has become a major focus area of various research institutes due to its ever-increasing prevalence worldwide. Maintaining a low level of blood glucose through impairing the activities of carbohydrate hydrolyzing enzymes, such as α-glucosidases has been among the major strategic approach in the management of T2DM. Hence, several drugs such as acarbose, voglibose, and miglitol that inhibit the activity of AGE have been developed and prescribed for people with T2DM [3,4]. However, these synthetic medicines were reported to have side effects and the search for plant-derived natural compounds with AGI activity have been gaining the interest of several research programs [5,6].

Metabolomics, which combines analytical chemistry and multivariate statistics, has become a preferred method used for the identification and quantification of bioactive compounds from natural products [1,7,8,9,10]. Metabolite profiling of pepper germplasms has shown the presence of diverse phytochemicals in the fruits [11,12,13]. Some of these metabolites have been associated with various health-promoting effects, i.e., antioxidant, anticancer, anti-inflammatory, antimicrobial, and antidiabetic effects [14,15,16,17].

Pepper is a member of Solanaceae family, which has been used as a source of nutrients in human diets. The fruit is the most economical part of the pepper plant, which has the characteristics of pungency, color, flavor, and taste. Biochemicals such as capsaicinoids, carotenoids, ascorbic acids, flavonoids, and other polyphenols that are found in the fruit are believed to be responsible for its unique characteristics [18,19,20]. Until today the fruit is the most studied part of the pepper plant compared to its leaves and few studies have reported the metabolite profiles and biological activities of pepper leaves. A study, for instance, by Maharijaya et al. [21] conducted untargeted metabolite characterization on a large number of different pepper leaves samples and reported more than 600 bioactive compounds. Antioxidant activity and polyphenols such as flavonoids and hydroxycinnamic acid derivatives including polyamines were also reported from the leaves of three pepper cultivars [17]. Isolates from the leaves of *Capsicum chinense* species have shown inhibitory activity against epoxide hydrolase [22]. Studies have shown the antidiabetic effect of pepper leaves extracts and some identified flavonoid glucosides with inhibitory activity against α-glucosidase [23,24]. However, most of the antidiabetic studies were conducted with few pepper samples except the work of Park et al. [25] and limited information is available about the relationship between metabolite profile and inhibitory activity of pepper leaves.

Hence, in this study, a large set of leaves of pepper germplasm (155) were investigated for their metabolic composition and antidiabetic activity. Chemometrics along with the bioactivity of extracts was implemented as a strategic approach for the discovery of natural compounds that can be used for the management of T2MD. Ultra-performance liquid chromatography (UPLC) coupled with quadrupole time-of-flight (QToF) mass spectrometry (MS) was used for the separation and identifications of targeted metabolites from pepper leaves. Multivariate statistics such as unsupervised principal component analysis (PCA), hierarchical clustering analysis (HCA), and supervised orthogonal partial least square analysis (OPLS) were employed for the combined analysis of antidiabetic activities and metabolomics data.

## 2. Results and Discussion

### 2.1. Identification of Polyphenols from Pepper Leaves Extracts

**Figure 1:**
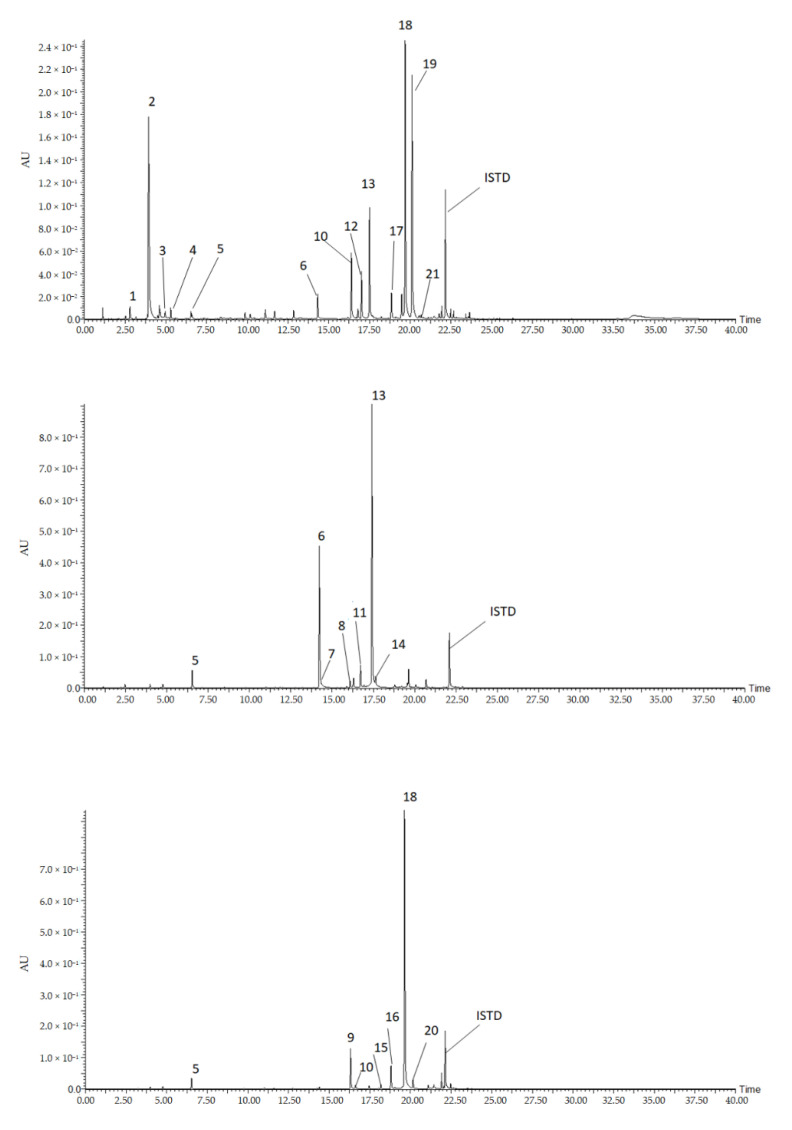
Representative chromatograms of the 21 detected compounds from pepper leaves extract. Compounds: (1), *N*-*cis* caffeoyl putrescine; (2), N-*trans* caffeoyl putrescine; (3), feruloyl putrescine; (4), coumaroyl putrescine; (5), 5 caffeoylquinci acid (5CQA); (6), luteolin 7-*O*-(2”-*O*-apiosyl)glucoside; (7), luteolin7-*O*-glucoside; (8), luteolin-*O*-(-apiosyl malonyl) hexoside; (9), apigenin 7-*O*-(2”-*O*-apiosyl)glucoside; (10), apigenin 7-*O*-glucoside; (11), luteolin-*O*-(apiosylmalonyl); (12), chrysoeriol-*O*-(apiosyl) hexoside; (13), Luteolin 7-*O*-(2”-*O*-apiosyl-6”-*O*-malonyl) glucoside; (14), luteolin-*O*-(malonyl) hexoside; (15), apigenin-*O*-(apiosylmalonyl) hexoside; (16), apigenin-*O*-(apiosylmalonyl) hexoside; (17), chrysoeriol-*O*-(apiosylmalonyl) hexoside; (18), apigenin 7-*O*-(2”-*O*-apiosyl-6”-*O*-malonyl) glucoside; (19), chrysoeriol-*O*-(-*O*-apiosylmalonyl) hexoside; (20), apigenin-*O*-(malonyl) hexoside; (21), chrysoeriol-*O*-(malonyl) hexoside.

In this study, we conducted targeted metabolomics for the identification of flavonoids and hydroxycinnamic acids conjugates from pepper leaves. Flavonoids are among the largest plant secondary metabolites that are synthesized by the phenylpropanoid metabolic pathway. Their structure contains a diphenyl propane backbone (C6-C3-C6) where a benzene ring (A) is attached with a pyrone ring (C), which is also substituted by a phenyl ring at its 2 or 3 positions [26]. More than 10,000 flavonoids have been reported from plants and they are mostly grouped into major sub-classes namely flavanones, flavones, isoflavones, flavonols, flavanols, and anthocyanidins [27]. Similarly, hydroxycinnamic acids (HCAs) and their conjugates are the other predominant plant phytochemicals synthesized via the phenylpropanoid pathway. Structurally, they contain C6-C3 backbone, and compounds such as caffeic acid, ferulic acid, sinapic acid, coumaric acid are the most common HCAs resulting from substitutions at the aromatic and aliphatic group. Moreover, HCAs conjugated with other compounds including amino acids, polyamines glycosides, etc., were identified from various plant sources [28,29,30]. In the following section, we presented the identification of flavonoids HCA derivatives, and hydroxycinnamic acid amides (HCAAs). The chromatographic condition separated the constituent of the pepper leaves extract as shown on the chromatogram (Figure 1). The structural identity of each peak was determined based on their retention time, MS fragments characteristics, UV absorbance, and literature data (Table 1).

#### 2.1.1. Hydroxycinnamic Acid Conjugates

Phenolamides and hydroxycinnamic derivatives were detected in pepper leaves. Coumaroyl, feruloyl, and caffeoyl substituted amines were the major HCAAs identified in pepper. A precursor ion [M+H]^+^ at *m*/*z* 251 was shown from compound **1** and **2**, which generated similar product ions upon further fragmentations. Both compounds produced fragments at *m*/*z* 163 with the loss of 88 da (putrescine (C_4_H_12_N_2_)) from the parent ions and with further ionization the product ions generated another fragment at *m*/*z* 135 due to the loss of CO (28 da). Hence, compounds **1** and **2** were assigned to be isomers and they were identified as N-cis-caffeoyl-putrescine and N-trans-caffeoyl-putrescine, respectively based on their elution order on a C18 column [28,31]. Previously, caffeoyl-putrescine was also reported from pepper leaves and fruits [17,25].

Compound 3 with its characteristic parent ion [M+H]^+^ at *m*/*z* 265 and product ion [M+H-88]^+^ at *m*/*z* 177 was preliminarily identified as feruloyl putrescine. Other fragmented ions due to loss of 32 da from feruloyl were observed at *m*/*z* 145. A molecular ion at *m*/*z* 235 was recorded from compound 4. ESI produced fragment ion at *m*/*z* 147 due to the loss of 88 da unit which was equivalent to the characteristics mass unit of coumaroyl moiety. The coumaroyl was further cleaved and exhibited an ion [M+H-88-CO]^+^ at *m*/*z* 119. Similar mass fragmentation products were reported from rice and compound 4 was tentatively identified as coumaroyl putrescine [31]. Compound 5 showed [M+H]^+^ at *m*/*z* 355 and daughter ion [M+H-C_7_H_10_O_5_]^+^ at *m*/*z* 181. Additional fragments were also observed from sequential loss of water and carbon monoxide molecules that generated [M+H-C_7_H_10_O_5_-H_2_O]^+^ at *m*/*z* 163 and [M+H-C_7_H_10_O_5_-H_2_O-CO]^+^ at *m*/*z* 135. Similar fragmentation patterns were reported by Wianowska and Gil (2019) [32] and compound 5 was identified as 5-*O*-caffeolyquinic acid. It was also detected in pepper [17,33], and other plants including, coffee, and thyme [34,35].

#### 2.1.2. Flavones

**Figure 2:**
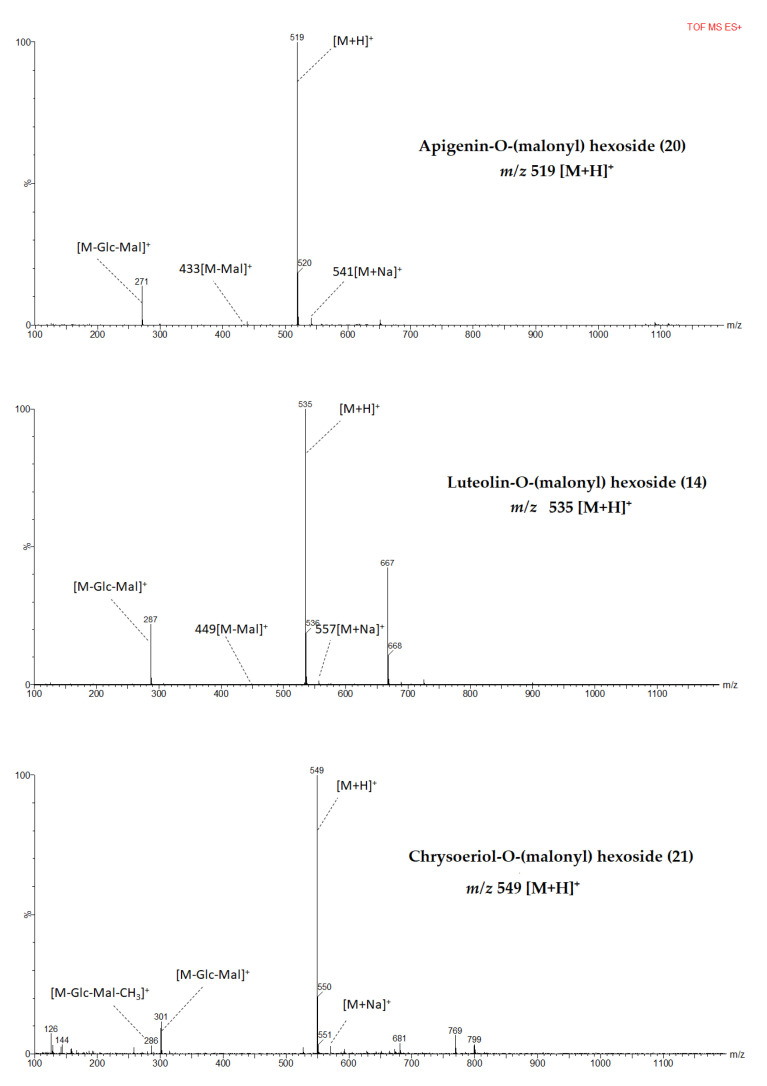
MS fragmentation patterns of metabolites identified from pepper leaf in positive mode ionization.

Identified flavones showed a characteristic fragment pattern obtained from cleavage of the O-glycosidic bonds between aglycone and sugar or between glycoside and malonyl moiety (Figure 2 and Figure S2). A total of 16 flavonoids that belong to apigenin, chrysoeriol, and luteolin derivatives were identified from pepper leaves extract.

##### Identification of Luteolin Derivatives

Compound 6 exhibited a molecular ion [M+H]^+^ at *m*/*z* 581, UV absorption maximum at 227,253, and 348 nm, and was described as luteolin 7-*O*-(2”-*O*-apiosyl) glucoside. The MS spectrum showed characteristics fragment ion at *m*/*z* 449 which resulted from the loss of one apiosyl unit (132 da) and a second product ion at *m*/*z* 287 [M+H-132-162 (glycosyl)]^+^. The compound has been identified from pepper leaves [17,24], pepper fruits [11,15,25,36,37] and celery [38,39]. An adduct ion [M+H]^+^ at *m*/*z* 449 was observed to co-elute at 14.39 min shouldered with compound 6 (14.32 min). A product ion [M+H-glycosyl]^+^ at *m*/*z* 287 indicated that compound **7** was luteolin 7-*O*-glucoside [17,22,24]. This compound has been isolated from pepper fruits [33], *Mentha x piperita* leaves [40], *Thymus schimperi, Thymus vulgaris,* [35,41] *Stachys lavandulifolia*, celery, and *Chrysanthemum* flowers [39,42,43].

Protonated molecular ion [M+H]^+^ at *m*/*z* 667 was detected from compounds **8**, **11**, and **13** which indicated that these compounds could be isomers. In addition, they showed the same fragments at *m*/*z* 535 [M+H-132]^+^ and 287 [M+H-132-162-86 (malonyl)]^+^. Among these isomers compound 13 was tentatively identified as luteolin 7-*O*-(2”-*O*-apiosyl-6”-*O*-malonyl) glucoside corresponding to major flavone glycoside in pepper leaves and fruits [24,37,44] and the result is consistent with previous findings from celery seed and leaves [39,45]. The other isomers, both compounds 8 and 11 could be assigned partially as luteolin-*O*-(apiosyl malonyl) hexosides and they are potential candidates to be identified as a new compound from the pepper leaves. Compound 14 with its molecular ion at *m*/*z* 535 and fragments at *m*/*z* 449 [M+H-86]^+^ and at *m*/*z* 287 [M+H-162-86]^+^ was tentatively identified as luteolin-*O*-(malonyl) hexoside. Previously, compound with similar characteristics has been identified as luteolin 7-*O*-(6”-*O*-malonyl) glucoside from *Calligonum azel*, *Syzygium* leaf, and *Chrysanthemum* flowers [43,46,47], however, to the best of our knowledge from a review of the literature, this is the first time that luteolin-*O*-(malonyl) hexoside reported from pepper leaves.

##### Identification of Apigenin Derivatives

Compound 9, showed an adduct ion [M+H]^+^ at *m*/*z* 565. Fragment ions at *m*/*z* 433 [M+H-apiosyl]^+^ and at *m*/*z* 271 [M+H-apiosyl-glycosyl]^+^ were observed and identified as apigenin 7-*O*-(2”-*O*-apiosyl)glucoside (apiin). Compound 10, was identified as apigenin 7-*O*-glucoside (cosmosin) which showed a protonated ion [M+H]^+^ at *m*/*z* 433 and fragment ion [M+H-162]^+^ at *m*/*z* 271. These two compounds were reported from pepper leaves [17,24], pepper fruits [33,36], celery seed and leaves [38,39] hops, and juniper berries [30], *Chrysanthemum* flowers, *Stachy landulifolia*, and chamomile flowers [42,43,48].

Compounds 15, 16, and 18 exhibited similar parent ions at *m*/*z* 651 [M+H]^+^ and product ions at *m*/*z* 535 [M+H-132]^+^ and *m*/*z* 271 [M+H-132-162-86]^+^. Compound 18 was tentatively identified as apigenin 7-*O*-(2”-*O*-apiosyl-6”-*O*-malonyl) glucoside which has been reported as a major flavone derivative from pepper leaves [24] while compound 15 and 16 were tentatively identified as apigenin-*O*-(apiosyl malonyl) hexosides. In another study, three isomers with similar fragment ions, elution order, and maximum absorption wavelengths were identified from parsley [49]. A molecular [M+H]^+^ at *m*/*z* 519 generated fragment ions at *m*/*z* 433 [M+H-86]^+^ and *m*/*z* 271 [M+H-86-162]^+^. Fragments pattern suggested that compound **20** was apigenin-*O*-(malonyl) hexoside which could be the first report from pepper leaves. However, previously a compound with a similar MS spectrum was identified as apigenin 7-*O*-(6”-*O*-malonyl) glucoside from flowers of *Chamomilla recutita* [48].

##### Identification of Chrysoeriol Derivatives

Compound 12 was partially identified as chrysoeriol-*O*-(apiosyl) hexoside with a molecular ion [M+H]^+^ at *m*/*z* 595. The parent ion yielded product ions [M+H-apiosy]^+^ at *m*/*z* 549 and [M+H-132-162]^+^ at *m*/*z* 301 which are the characteristics fragments of chrysoeriol 7-*O*-(2”-*O*-apiosyl) glucoside as widely reported from *Angelica keiskei*, *Calligonum azel*, purple radish, *Lycium ruthenicum*, *Syzygium* leaf, *Rhamnus petiolaris* [45,46,47,50,51,52]. In this study, chrysoeriol derivatives were detected in less than 12% of the pepper genotypes.

Compounds 17 and 19 showed the same protonated molecular ion [M+H]^+^ at *m*/*z* 681. The product ions at *m*/*z* 549 [M+H-apiosyl]^+^ and at *m*/*z* 301 [M+H-apiosyl-glycosyl-malonyl]^+^ were observed from both compounds.

Hence, compounds 17 and 19 were tentatively identified as isomers of chrysoeriol-*O*-(apiosyl malonyl) hexoside. A compound, with similar molecular ion and MS fragments, has been reported as chrysoeriol 7-*O*-(malonyl apisoyl) glucoside from parsley, Syzygium leaf, celery, and hawthorn [39,52,53,54]. Compound 21 which was assigned to be chrysoeriol-*O*-(malonyl) hexoside was tentatively identified based on its molecular ion [M+H]^+^at *m*/*z* 549 and fragment ions [M+H-glycosyl-malonyl]^+^ at *m*/*z* 301 [38]. This is the first report on chrysoeriol-*O*-(malonyl) hexoside identification from pepper leaves according to the best of our knowledge from a review of the literature.

### 2.2. Flavones Decorations/Modifications

Glycosylation of the hydroxyl groups in the A, B, and C rings of flavonoids and acylation of sugar moieties are the most common modifications observed in plant flavonoid biosynthesis [55]. These modifications are known to enhance the bioactivity, solubility, and stability of the corresponding aglycones in the plant system, and they are mostly catalyzed by the glycosyltransferase and acyltransferase enzymes, respectively [3,56,57]. The flavones derivatives identified in this study showed similar glycosylation and acylation pattern in all investigated pepper genotypes (Table 1 and Figure S1). This suggested a conserved flavone modification pathway in pepper leaves. However, genetic variation at the core of flavones biosynthetic pathways might be responsible for the presence of different glycosylated and acylated aglycones compositions (apigenin, luteolin, and chrysoeriol) across the genotypes. Luteolin and chrysoeriol are synthesized from apigenin and luteolin, respectively, showing a linear biosynthetic relationship between them [58,59,60].

In plants glycosylation or acylation are usually the final steps in the flavonoid biosynthesis pathway [55]. For example, apigenin-*O*-(apiosyl malonyl) hexoside identified here, could have been derived from acylation of apigenin 7-*O*-(2”-*O*-apiosyl) glucoside or glycosylation of apigenin 7-*O*-(6”-*O*-malonyl) glucoside. Therefore, these two separate modifications could be the reason for the presence of isomers of the malonated diglycosides of apigenin, luteolin, and chrysoeriol in pepper leaves [61]. The isomers might have resulted from different glycosylation positions of the flavones, or malonylation could have occurred at various positions of the glucose or apiosyl. The 7-*O* position of the flavones and 6″-position glucose has been reported as the most frequent site of glycosylation and acylation, respectively [62,63]. For example, formononetin glucoside malonate isomers were found to show two acylation points on the sugar group identified as 7-*O*-β-d-glucoside 6”-*O*-malonate and 7-*O*-β-d-glucoside 4”-*O*-malonate [64]. Moreover, the position of the glycosidic substitute on the aglycone was reported to determine the elution order of glycosylated flavonoids. For instance, luteolin 5-*O* glucoside was found to elute earlier than luteolin 7-*O* glucoside [65]. Similarly, the substitution of glucose by galactose moiety was found to shorten the retention time of flavonoids [62]. Therefore, the observed isomers might be containing a galactose moiety, or 5-*O* glycosylation and/or 4”-*O*-malonate. However, the exact structure of conjugation could not be determined with only MS spectrometry and it requires the use HNMR analysis.

### 2.3. Relationship between AGI Activity and Metabolite Profile of Pepper Leaves

#### 2.3.1. Evaluation of AGI Activity of the Pepper Leaf Extracts

**Figure 3:**
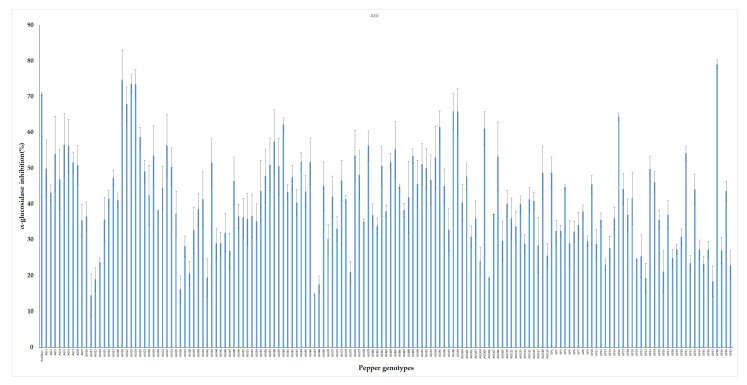
In vitro α-glucosidase inhibitory activities of pepper leaf extracts. The result is presented as mean ± SD (n = 3).

Plant extracts possess metabolites that are known to participate in a range of health-promoting physiological processes. In vitro inhibitory potential of 155 pepper leaf extracts were screened against yeast α-glucosidase enzyme. Pepper genotypes showed wide variation in their antidiabetic potentials. AGI activities obtained from the leaf extracts ranged from 17.15% to 79.1% with an average value of 44.83%. The highest inhibition activity was obtained from genotype GP38 while low AGI activities were observed from genotypes such as AGI12, GP21, and GP37 (Figure 3). Acarbose was used as positive control and it showed an average of 70.8% AGI activity. This result agrees with previous studies on pepper and other plant samples. Pepper leaves harvested at different stages i.e., April, July, and October were found to show an average of 36.18%, 66.61%, and 47.29% AGI activities, respectively [66]. Similarly, Kim et al. reported that leaves extracts of pepper exhibited 61.1% α-glucosidase inhibition [24]. Fruit extract from a red sweet pepper type exhibited 57% inhibitory potential against yeast α-glucosidase enzymes [67]. Plants including okra, bitter gourd, radish, asparagus, sweet potato, and grape were also reported to possess antidiabetic properties [8,68,69,70,71,72].

**Figure 4:**
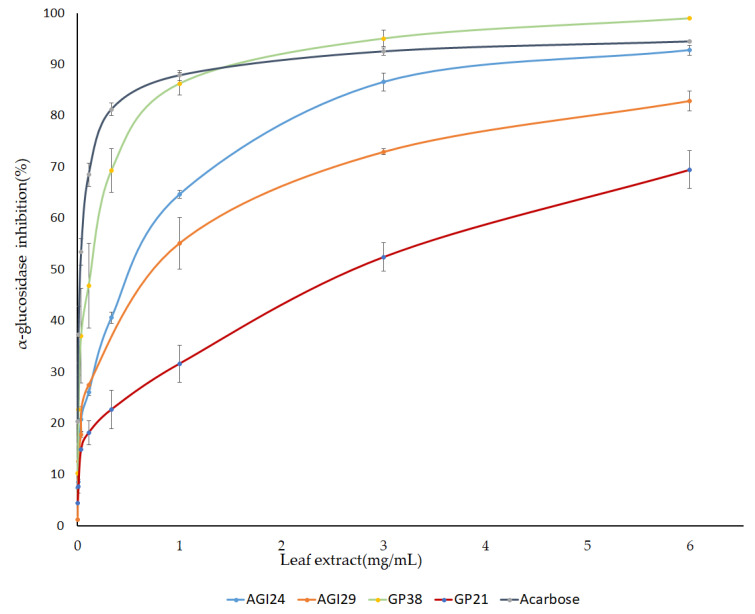
IC_50_ values of pepper leaf extracts and acarbose against α-glucosidase enzyme. The result is presented as mean ± SD (n = 3).

Furthermore, a dose-dependent inhibition assay was performed using PLEs of selected genotypes. Pepper leaf extracts of three genotypes namely AGI24, AGI29, and GP38 that showed high AGI activity, and one genotype (GP21) with low AGI activity were prepared at varying concentrations and their IC_50_ was calculated from the generated dose curves (Figure 4). “Genotypes with high AGI activity showed IC_50_ values ≥ 0.5 mg/mL”. Interestingly, the genotype GP38 with IC_50_ value of 0.122 mg/mL was found to be more potent than acarbose (IC_50_ = 0.19 mg/mL) (Table 2). This indicated that pepper leaves are a promising source of natural α-glucosidase inhibitors.

#### 2.3.2. Multivariate Analysis

**Figure 5:**
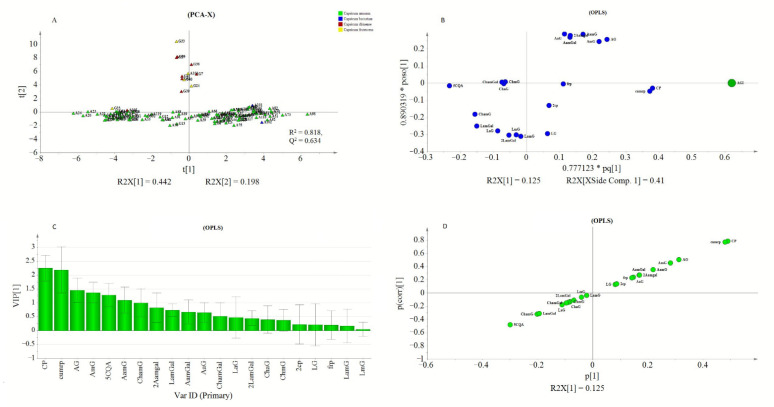
PCA score plot showing pepper genotypes: (**A**) OPLS loading plot (**B**) and VIP plot (**C**) showing the contribution of metabolites to the AGI activity, and S-plot (**D**).

Peak areas of detected metabolites and AGI activity values obtained from PLE were subjected to rank inverse transformation, unit variance scaling, and mean centering before they were used for multivariate analysis using SIMCA P^+^. PCA was conducted to assess the classification or grouping of genotypes based on their metabolite signatures. Orthogonal partial least square (OPLS) was used to investigate the relationship between the detected metabolite profiles and the measured AGI activity of the PLEs. In the PCA analysis, PC1 and PC2 explained 44.2% and 19.8% of the total variations, respectively. In (Figure 5A), three major groupings of the genotypes were observed. The first two groups were located towards the positive and negative axes of PC1 while the third group was located on the positive side of the PC2. The values of R^2^X and Q^2^ showed the adequacy of the PCA model in grouping the genotypes (Figure 5A). Hence, OPLS analysis was performed to extract AGI activity-related variations from those that are orthogonal to the response variable

**Figure 6:**
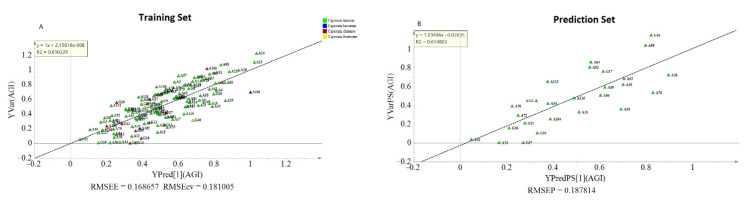
Observed and predicted AGI activities of the training set (**A**); prediction plot using separate test samples (**B**).

The OPLS model exhibited a cumulative R^2^Y and Q^2^ value of 0.61 and 0.546, respectively. The observed R^2^ and Q^2^ values could be taken as fair indicators of the model’s adequacy [73]. Moreover, the root means squared error of estimation (RMSEE) at 16.8 and root mean squared error of cross-validation (RMSEcv) at 18.1 exhibited a good fit and prediction capacity of the OPLS model, respectively. To assess the prediction capacity, the OPLS regression model was used to predict AGI activities from a test set of samples that were not included in the first OPLS analysis. As shown in Figure 6B the scores approached the ideal diagonal line (R^2^ = 0.615) with acceptable root mean squared error of prediction (RMSEP) at 18.7. Cross-validation with ANOVA showed a statistically significant OPLS model with a *p*-value of 1.32 × 10^−19^ (Table 3).

In the loading plot (Figure 5B), CP and CumP were located far from the center but closer to the AGI point. Most of the apigenin derivatives (1st quadrant) and luteolin derivatives (3th quadrant) were found to show strong weight on the orthogonal components. The proximity of metabolites to the response variable (AGI), could indicate their higher contribution to the observed AGI activities. Metabolite’s contributions to AGI activity were also reflected by the VIP scores and S-plot. The CP and cumP showed a VIP score that was >2 (Figure. 5C) and those metabolites with VIP value ≥1 could be considered as key predictors for bioactivity [74]. Similarly, in the S-plot, CP, and cumP were placed far from the origin indicating their high correlation to the measured AGI activities of pepper leaves extracts. Previously various studies have applied chemometrics for the identification of bioactive compounds from natural sources [10,75,76,77].

Model validation using cv-ANOVA and prediction test showed that the model is fairly adequate to explain the AGI response based on the metabolite composition of pepper leaves. However, only 12% of the total variation was related to AGI activity while the rest of the variations were due to metabolic differences among the genotypes. This difference in metabolite composition hindered the development of a more adequate OPLS regression model, which might result from inconsistent co-variation between AGI activity and metabolite compositions. Hence, hierarchical clustering analysis (HCA) was conducted based on the PCA model (Figure 5A), and the generated three clusters (Figure S3). were used to conduct a new analysis using the OPLS-Class model.

The adequacy of the model could be increased using a separate OPLS-Class analysis for each cluster. As displayed in Table 4, R^2^ and Q^2^ values obtained from each class were higher than their previous estimations from the OPLS model. For instance, the estimated R^2^ and Q^2^ in class-1. were 0.856 and 0.775, respectively, showing improved goodness of fit and prediction capacity. Interestingly, most of the genotypes found in this class were double haploid lines that were derived from crosses of the same parents which could account for the similarity observed in their metabolite composition pattern. Likewise, the two clusters showed an increased R^2^Y and Q^2^ values.

**Figure 7:**
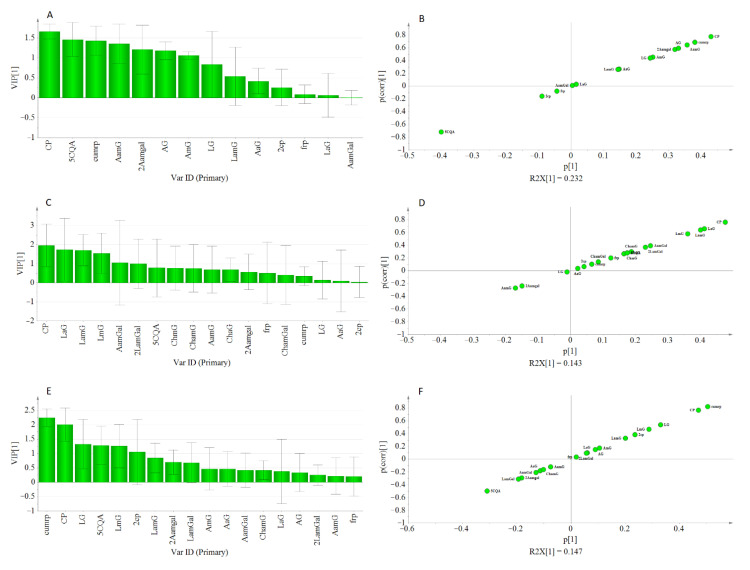
Displayed VIP Plot, and S-plot from OPLS-class analysis showing the contribution of metabolites to the AGI activity in class-1 (**A**,**B**), class-2 (**C**,**D**), and class-3 (**E**,**F**).

Moreover, in each cluster, the VIP scores for caffeoyl-putrescine were ≥1.5, and other metabolites that showed lesser VIP scores in the OPLS model were found to show higher values under the OPLS-Class model analysis (Figure 7). Hence, it might be crucial to consider the pattern of metabolites variations among genotypes when metabolomics and bioassay-guided screening is used for the identification of bioactive compounds from large sample sets.

### 2.4. AGI Activity from Pure Standard Compounds

**Figure 8:**
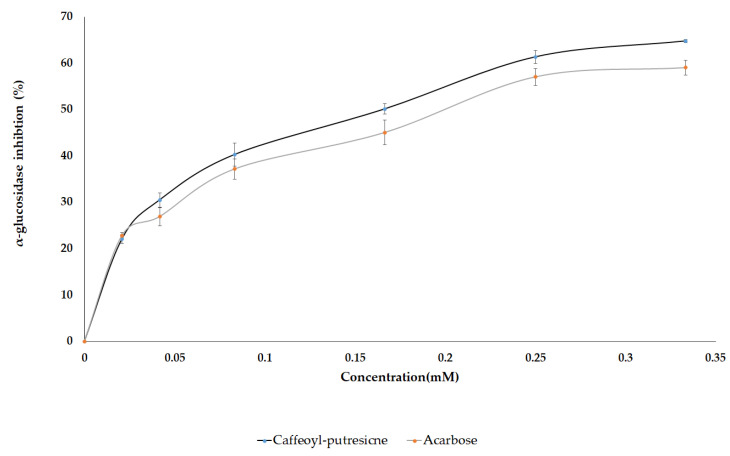
Dose-dependent inhibition of α-glucosidase enzyme using the caffeoyl-putrescine and acarbose.

In several in vitro studies, AG and LG were reported to exhibit inhibitory activity against the α-glucosidase enzyme. However, limited information is available on the antidiabetic potential of the diglycoside of flavones as well as CP which were identified in this study. Therefore, based on the VIP scores, and the metabolites abundance in pepper leaves extract, the pure standard compounds of CP, AG, LG, AaG, and LaG were assayed against yeast α-glucosidase enzyme to assess their anti-diabetic effect. Moreover, luteolin, apigenin, and acarbose (a positive control) were included for comparison. Among the standard compounds, representing the identified metabolites, caffeoyl putrescine was found to be the most potent compound that showed a dose-dependent inhibition against the enzyme with an IC_50_ value of 145 µM while the amount required for acarbose was 197 µM (Figure 8 and Table 3). The result was consistent with the above OPLS analyses as it was reflected by the VIP score of the selected metabolites. Studies have shown other health-promoting effects of polyamines identified from plants. They exhibit antioxidant, anti-inflammatory, anti-cancer, anti-obesity, and neuroprotective properties [78]. Putrescine conjugates such as *N*-*p*-coumaroyl-*N*′-feruloyl putrescine and *N*,*N*′-diferuloyl putrescine were reported as potent α-glucosidase inhibitors [79]. Similarly, kukoamines A and kukoamines B exhibited antidiabetic effects in the mice model [78,80]. However, this is the first report on in vitro α-glucosidase inhibition using caffeoyl putrescine.

Mono and diglycosides of apigenin and luteolin were found to be less potent than CP. However, their aglycone forms, luteolin, and apigenin were strong inhibitors with an IC_50_ value of 7.6 and 81 µM, respectively (Table 3). According to an earlier study, the increase in molecular size and change in polarity due to glycosylation was considered as the possible causes for the observed reduction in inhibitory effect [3]. Apigenin 7-*O*-glucoside and luteolin 7-*O*-glucoside, isolated from *Syzygium cumini* Linn. seeds and pepper leaves, respectively, were found to exhibit inhibitory activity against α-glucosidase [23,81]. The poor AGI activities observed from apiin and LaG were in agreement with the work reported by Kim et al. [24]. In the report, separate fractions of pepper leaves extract that was rich in apiin and LaG were found to show very low AGI activity. On the other hand, studies showed the hydrolysis of food flavonoids through the activity of *β*-glucosidase which converts flavonoid glycosides into their aglycone forms [82]. For instance, human small intestinal *β*-glucosidase was shown to deglycosylate the mono and diglycosides of quercetin [83]. Therefore, through possible synergetic action of caffeoyl putrescine and produced flavone aglycones (apigenin and luteolin) against α-glucosidase, pepper leaves could provide improved antidiabetic effects in the human body. However, this hypothesis on pepper leaves needs to be supported with an in vivo experiment.

## 3. Materials and Methods

### 3.1. Chemicals and Reagents

Standards of 6-methoxyluteolin, apigenin, luteolin, luteolin7-*O*-glucoside, apigenin7-*O*-glucoside were purchased from Extrasynthese (Lyon, Genay Cedex, France); apiin, luteolin7-*O*-(2”-*O*-apiosyl)glucoside, caffeoyl-putrescine from ALB Technology(Hong Kong); 4-nitrophenyl-β-D-glucopyranoside (pNPG), α-glucosidase (yeast, EC 3.2.1.20), acarbose, sodium carbonate from Sigma Aldrich Co. (St. Louis, MO, USA); sodium phosphate buffer from Biosesang (Seongnam, Korea); methanol from MERCK (Damstadt, Germany); acetonitrile from Fisher Scientific (Fair Lawn, NJ, USA); and formic acid from Junsei Chemical (Tokyo, Japan).

### 3.2. Pepper Plant Materials

A total of 155 Pepper breeding materials and germplasms representing different *Capsicum* species i.e., *C. annuum*, *C. frutescence*, *C. chinense*, and *C. baccatum* were grown in 2019 (February to July). Sixty days after sowing, four healthy seedlings per each accession were transplanted in a greenhouse at the vegetable research station of the National Institute of Horticultural and Herbal Science, Rural Development Administration, Korea. Fully grown young pepper leaves were harvested at three months after transplanting and immediately kept at −80 °C for one day before they were lyophilized using Freeze Dryer (IlShin BioBase, Ilshin Lab Co., Ltd., Dongducheon, Korea).

### 3.3. Preparation of Leaf Extracts

Extraction was carried out using the previous method [84], with a minor modification. In brief, dried pepper leaves were grounded using a blender and 500 mg of powder was mixed with 5 mL of extraction solvent (methanol:water:formic acid, 50:45:5). The solution was vortexed for 30 s, sonicated using ultrasonic cleaner (UUIL Ultrasonic Co. Ltd., Ansan, Korea) for 25 min at 35 °C and 30 Hz, centrifuged at 3500 rpm for 15 min at 4 °C, and the supernatant was filtered using column PD-10 (GE Healthcare, Buckinghamshire HP7 9NA, UK). Finally, the extraction solvent was removed in EYELA evaporator (Tokyo Rikakikai Co., Ltd., Tokyo, Japan), and the crude extract was lyophilized and stored at −80 °C until further analysis.

### 3.4. Solid-Phase Extraction (SPE)

Pepper leaves extract (PLE) weight was measured, re-dissolved in the extraction solvent to a final concentration of 10 mg/mL, and filtered using 0.2 µm syringe filter, polyvinylidene fluoride (PVDF, Hyundai micro, Seoul, Korea). The SPE was done using Hypersep SPE 500 mg/2.8 mL C18 (Thermos scientific, 197 Cardiff Road, Rockwood TN 37854, USA) and vacuum manifold processing station (Agilent technologies, Santa Clara, CA, USA), following the steps: activation, 2.5 mL of methanol, conditioning, 5 mL water, sample loading 0.5 mL of the filtered extract dissolved in 4.5 mL water; internal standard loading, 0.5 mL of 6-methoxyluteolin (50 ppm) dissolved in 4.5 mL water; washing, 5 mL water; elution, 5 mL methanol containing 1% formic acid). Finally, the elute was concentrated using N_2_ gas (EvaN-0600) and re-dissolved in 500 µL extraction solvent, and injected into UPLC-DAD-QToF-MS.

### 3.5. Identification of Phenolic Compounds Using UPLC-DAD-QToF-MS

Flavonoids and phenolic acids in pepper leaves extract were separated on a Waters UPLC with C18 column (CORTECS^®^ UPLC^®^ T3; 2.1 × 150 nm I.D., 1.6 μm) coupled with a diode array detector (Waters Co., Milford, MA, USA). Separations were performed with a binary solvent system under the following chromatographic setup: mobile phases, water with 0.5% formic acid (**A**) and acetonitrile containing 0.5% formic acid (**B**); column oven temperature, 30 °C; flow rate, 0.3 mL/min; injection volume, 1 μL. The elution program was as follows: 0–20 min, 5−20% B; 20 min, 20–25% B; 25–30 min, 25–50% B; 30–32 min, 90% B; and 35–40 min, 5% B. Absorbance for flavonoids were recorded at 350 nm and phenolic acids were recorded at 320 nm. Metabolites mass was determined using a quadrupole time-of-flight mass spectrometry (Xevo G2-S QTOF-MS; Waters Micromass, Manchester, UK) with electrospray ionization source in the following settings: positive mode Ionization, capillary voltage 3.5 kV, desolvation gas, 1020 L/h, sample cone voltage, 40 V, source temperature, 120 °C, and desolvation temperature, 500 °C. The mass to charge (*m*/*z*) ratios of parent and product ions were recorded in the range of 100 to 1200 *m*/*z*. Data acquisition and processing were conducted using the MassLynx V4.1 software (Waters, Milford, MA, USA). Retention time, UV spectra, and mass fragmentation pattern of metabolites were compared with an in-house prepared library and results of published reports for the tentative identification of the compounds.

### 3.6. Yeast α-Glucosidase Inhibition (AGI) Assay

Inhibitory activity of pepper leaves extracts(PLE) was determined according to Kim et al. [27] with modifications. Leave extract, substrate, enzyme, and acarbose solution were prepared in 0.1 M sodium phosphate buffer. The assay was conducted on 96 well microplates as follows: 50 µL of PLE (6 mg/mL) was mixed with 200 µL of yeast enzyme (0.1 U/mL) and incubated at 37 °C for 10 min. Then 50 µL of pNPG substrate (3 mM) was added into the mixture and kept at 37 °C for another 15 min. Finally, the reaction was stopped using 2 mL of 0.1 M Na_2_CO_3_. Furthermore, pepper genotypes that showed high or low AGI activity were selected and their PLE were prepared at a range of concentrations (0.025, 0.075, 0.222, 0.666, 2, 6, 18, 36 mg/mL). Their inhibition activity was obtained according to the above protocol and the dose-response curve was used to calculate the concentration that showed 50% inhibition (IC_50_). Acarbose was used as a positive control at 15 mM.

Additionally, pure standard compounds representing some of the metabolites identified from pepper leaves and acarbose were dissolved in 0.1 M phosphate buffer containing 50% DMSO prepared to give final concentrations ranging from 5.2 µM to 333 µM in the reaction well. Here, the inhibitory assay reported by Yao et al. [85] was used with some changes. Briefly, the pure compounds and enzyme solutions (0.025 U/mL) were prepared in 96 well microplates. The plates were kept at 37 °C for 10 min and pNPG (3 mM) was added to start the reaction. The enzymatic reaction was allowed to continue for another 10 min before it was stopped using 0.1 M Na_2_CO_3_ solution. The amount of 4-nitrophenol generated from the reaction was measured at 405 nm and its absorbance value obtained from control and sample wells were used to measure the AGI activity. Enzyme inhibition activity of pepper extracts and pure compounds were determined spectrophotometrically by measuring the absorbance of the produced 4-nitrophenol at 405 nm in a microplate reader.

Absorbance from the control (where no inhibitors were added) represented maximum enzyme activity. Another set of mixtures prepared with PLE/standard substrate, and 0.1 M phosphate buffer (instead of enzyme) was used as sample blank. Buffer was used as a blank for the instrument.

### 3.7. Statistical Analysis

Metabolite data and AGI values were collected from three replicates and presented as mean ± standard deviation (SD). The peak area of each metabolite was normalized using the average peak area of the internal standard. All data including AGI activity values collected from each genotype were subjected to rank-inverse transformation using SPSS (ver. 16.0, SPSS Inc.). Multivariate statistical analyses such as unsupervised principal component analysis (PCA), hierarchical clustering analysis (HCA), orthogonal partial least square analysis (OPLS), and OPLS-Class analysis were carried out using SIMCA-P software (ver. 14.0, Umetrics, Umeå, Sweden). IC_50_ values of PLE and pure standard were calculated from the log fitted response dose curve.

## 4. Conclusions

In summary, our study demonstrated that flavone derivatives were the major forms of flavonoid presented in pepper leaves that might have been synthesized by a conserved modification/decoration pathway across the genotypes. The leaf extract of pepper (*Capsicum* spp.) was confirmed as a potential α-glucosidase enzyme inhibitor. For the first time, the result found out caffeoyl-putrescine, as a potent inhibitor of carbohydrate hydrolyzing enzyme which can be used for the management of T2DM. Hence, the combined application of chemometrics with high throughput screening of AGI activity is an effective approach for the discovery of potential potent α-glucosidase inhibitors from a large set of genotypes.

## Figures and Tables

**Table 1:** Name, retention time, molecular ion, fragment ions, and adduct ions of metabolites identified from leaf extract of pepper (*Capsicum* spp.).

No.	Compounds Name	RT (min)	UVλ_max_ (nm)	MolecularWeight	Molecular Formula	Experimental(*m*/*z*) [M+H]^+^,[M+Na]^+^,[M+K]^+^	Fragmentations (*m*/*z*)
1	N-*cis* caffeoyl putrescine	2.82	230	250	C_13_H_18_N_2_O_3_	251,273,ND	163,135
2	N-*tans* caffeoyl putrescine	3.95	230,318	250	C_13_H_18_N_2_O_3_	251,273,ND	163,135
3	Feruloyl putrescine	4.97	227,230	264	C_14_H_20_N_2_O_3_	265,277,ND	177,145
4	Coumaroyl putrescine	5.33	230,292	234	C_13_H_18_N_2_O_2_	235,257,ND	147,119
5 ^a^	5 caffeoylquinci acid (5CQA)	6.59	231,325	354	C_16_H_18_O_9_	355,377,393	337,181,163,145,135
6 ^a^	Luteolin 7-*O*-(2”-*O*-apiosyl)glucoside	14.32	227,253,348	580	C_26_H_28_O_15_	581,603,619	449,287
7 ^a^	Luteolin 7-*O*-glucoside	14.39	227,347	448	C_21_H_20_O_11_	449,471,ND	287
8 ^b^	Luteolin-*O*-apiosyl malonyl). Hexoside (isomer of 13)	16.18	227,348	666	C_21_H_20_O_11_	667,689,705	535,287
9 ^a^	Apigenin 7-*O*-(2”-*O*-apiosyl)glucoside	16.38	228,266,337	564	C_26_H_28_O_14_	565,587,603	433,271
10 ^a^	Apigenin 7-*O*-glucoside	16.69	227,266,336	432	C_21_H_20_O_10_	433,455,ND	271
11 ^b^	Luteolin 7-*O*-(2”-*O*-apiosyl-6”-*O*-malonyl)glucosideLuteolin-*O*-(apiosyl malonyl) hexoside (isomer of 13)	16.8	227,348	666	C_29_H_30_O_18_	667,689,705	535,287
12	Chrysoeriol-*O*-(apiosyl) hexoside	17.01	227,347	594	C_27_H_30_O_15_	595,617,633	463,301,286
13	Luteolin 7-*O*-(2”-*O*-apiosyl-6”-*O*-malonyl)glucoside	17.48	227,254,348	666	C_29_H_30_O_18_	667,689,705	535,287
14 ^b^	Luteolin-*O*-(malonyl) hexoside	17.71	227.348	534	C_24_H_22_O_14_	535,557,ND	449,287
15 ^b^	Apigenin-*O*-(apiosyl malonyl) hexoside(isomer of 18)	18.23	227,336	650	C_29_H_30_O_17_	651,673,689	519,271
16 ^b^	Apigenin-*O*-(apiosyl malonyl) hexoside (isomer of 18)	18.85	227,267,337	650	C_29_H_30_O_17_	651,673,689	519,271
17 ^b^	Chrysoeriol-*O*-(apiosyl malonyl) hexoside (isomer)	19.5	227,347	680	C_30_H_32_O_18_	681,703,719	549,301,286
18	Apigenin 7-*O*-(2”-*O*-apiosyl-6”-*O*-malonyl)glucoside	19.67	230,267,336	650	C_29_H_30_O_17_	651,673,689	519,271
19	Chrysoeriol-*O*-(apiosyl malonyl) hexoside (isomer)	20.1	227,251,346	680	C_30_H_32_O_18_	681,703,719	549,301,286
20 ^b^	Apigenin-*O*-(malonyl) hexoside	20.2	227,267,336	518	C_24_H_22_O_13_	519,541,ND	433,271
21 ^b^	Chrysoeriol-*O*-(malonyl) hexoside	20.62	231,347	548	C_25_H_24_O_14_	549,571,ND	301,286

^a^ Further confirmed in comparison with authentic standards. ^b^ New compounds tentatively identified in pepper leaves. ND-not determined.

**Table 2:** α-Glucosidase inhibitory activities of pure standard compounds and leave extracts of selected pepper genotypes.

Pepper Extract ^a^				
Genotypes	IC_50_ (mg/mL)	Standard Compounds ^b^	Inhibition% (250 µM)	IC_50_ (µM)
A24	0.34 ± 0.04	Caffeoyl-Putrescine	61.36 ± 1.37	145
A29	0.5 ± 0.035	Acarbose	57.06 ± 1.8	197
G38	0.122± 0.004	Apigenin-7-*O* glucoside	30.68 ± 5.35	>300
G21	2.5 ± 0.22	Luteolin-7-*O* glucoside	37.65 ± 3.08	>300
ACARBOSE	0.19 ± 0.034	Apiin	13.25 ± 0.3 ^d^	ND
		Luteolin-7-*O*-(2” apiosyl)glucoside	29.9 ± 4.02 ^d^	ND
		Luteolin	97.84 ± 0.56 ^c^	7.6
		Apigenin	56.14 ± 2.37 ^c^	81

^a^ Inhibition was conducted according to Y. C. Kim, Choi, Lee, and Lee, 2018) (enzyme concentration 0.1 U/mL, 3 mM pNPG). ^b^ Inhibition was conducted according to (Yao, Cheng, Wang, Wang, and Ren, 2011 (enzyme concentration 0.025 U/mL, 3 mM pNPG). ^c^ Inhibition obtained at 82 µM concentration. ^d^ Inhibition obtained at 500 µM concentration. ND = not determined.

**Table 3:** cv ANOVA from cross-validation analysis of the OPLS model.

OPLS Model	SS	DF	MS	F	*p*	SD
Total	125	125	1			1
Regression	67.5143	4	16.8786	35.5272	1.32 × 10^−19^	4.10835
Residual	57.4857	121	0.475089			0.689267

**Table 4:** Measures of multivariate analysis model’s fitness and prediction power.

Model	Type	Components	N	R^2^X (cum)	R^2^Y (cum)	Q^2^ (cum)
M1	PCA-X	4	126	0.818	-	0.634
M2	OPLS	1 + 1 + 0	126	0.535	0.61	0.54
M3	OPLS-Class (1)	1 + 2 + 0	39	0.578	0.856	0.775
M4	OPLS-Class (2)	1 + 3 + 0	11	0.753	0.945	0.565
M5	OPLS-Class (3)	1 + 2 + 0	76	0.544	0.726	0.605

## Data Availability

The raw data supporting the findings presented in this study are available on request from the corresponding author.

## Supplementary Materials

The following are available online at https://www.mdpi.com/article/10.3390/metabo11100649/s1, Figure S1: Relative abundance of selected pepper leaf’s metabolites across 155 genotypes, and Figure S2: Mass fragmentation and chemical structure of Chrysoeriol-*O*-(apiosyl) hexoside (**A**) Luteolin 7-*O*-(2”-*O*-apiosyl) glucoside (**B**) and Apigenin 7-*O*-(2”-*O*-apiosyl) glucoside (**C**), Figure S3: Dendrogram constructed from the PCA model showing three major clusters, Figure S4: Mass fragmentation and chemical structure of apigenin-*O*-(apiosyl malonyl) hexoside (**A**) and its isomers (**B**) and (**C**), Figure S5: Mass fragmentation and chemical structure of luteolin (apiosyl malonyl) hexoside (**A**) and its isomers (**B**) and (**C**), Figure S6: Mass fragmentation and chemical structure of chrysoeriol (apiosyl malonyl) hexoside (**A**) and its isomer (**B**).

## Abbreviations

5CQA	5 caffeoylquinic acid
2cp	N-*cis* Caffeoyl putrescine
CP	N-*tans* Caffeoyl putrescine
cumrp	Coumaroyl putrescine
frp	Feruloyl putrescine
AaG	Apigenin 7-*O*-(2”-*O*-apiosyl) glucoside
AamGal	Apigenin-*O*-(apiosyl malonyl) hexoside
2AamGal	Apigenin-*O*-(apiosyl malonyl) hexoside
AamG	Apigenin 7-*O*-(2”-*O*-apiosyl-6”-*O*-malonyl) glucoside
AmG	Apigenin-*O*-(malonyl) hexoside
AG	Apigenin 7-*O*-glucoside
ChmG	Chrysoeriol-*O*-(malonyl) hexoside
ChaG	Chrysoeriol-*O*-(apiosyl) hexoside
ChaGal	Chrysoeriol-*O*-(apiosyl malonyl) hexoside
ChamG	Chrysoeriol-*O*-(apiosyl malonyl) hexoside
LaG	Luteolin 7-*O*-(2”-*O*-apiosyl) glucoside
LamGal	Luteolin-*O*-(apiosyl malonyl) hexoside
2LamGal	Luteolin-*O*-(apiosyl malonyl) hexoside
LamG	Luteolin 7-*O*-(2”-*O*-apiosyl-6”-*O*-malonyl) glucoside
LmG	Luteolin-*O*-(malonyl) hexoside
LG	Luteolin 7-*O*-glucoside
AU	Absorbance unit
