# Supplementary figures and images for: Identification of α-Glucosidase Inhibitors from Leaf Extract of Pepper (Capsicum spp.) through Metabolomic Analysis
Source: Metabolites. 2021 Sep 22;11(10):649. doi: 10.3390/metabo11100649 (PMC8538662; doi:10.3390/metabo11100649)

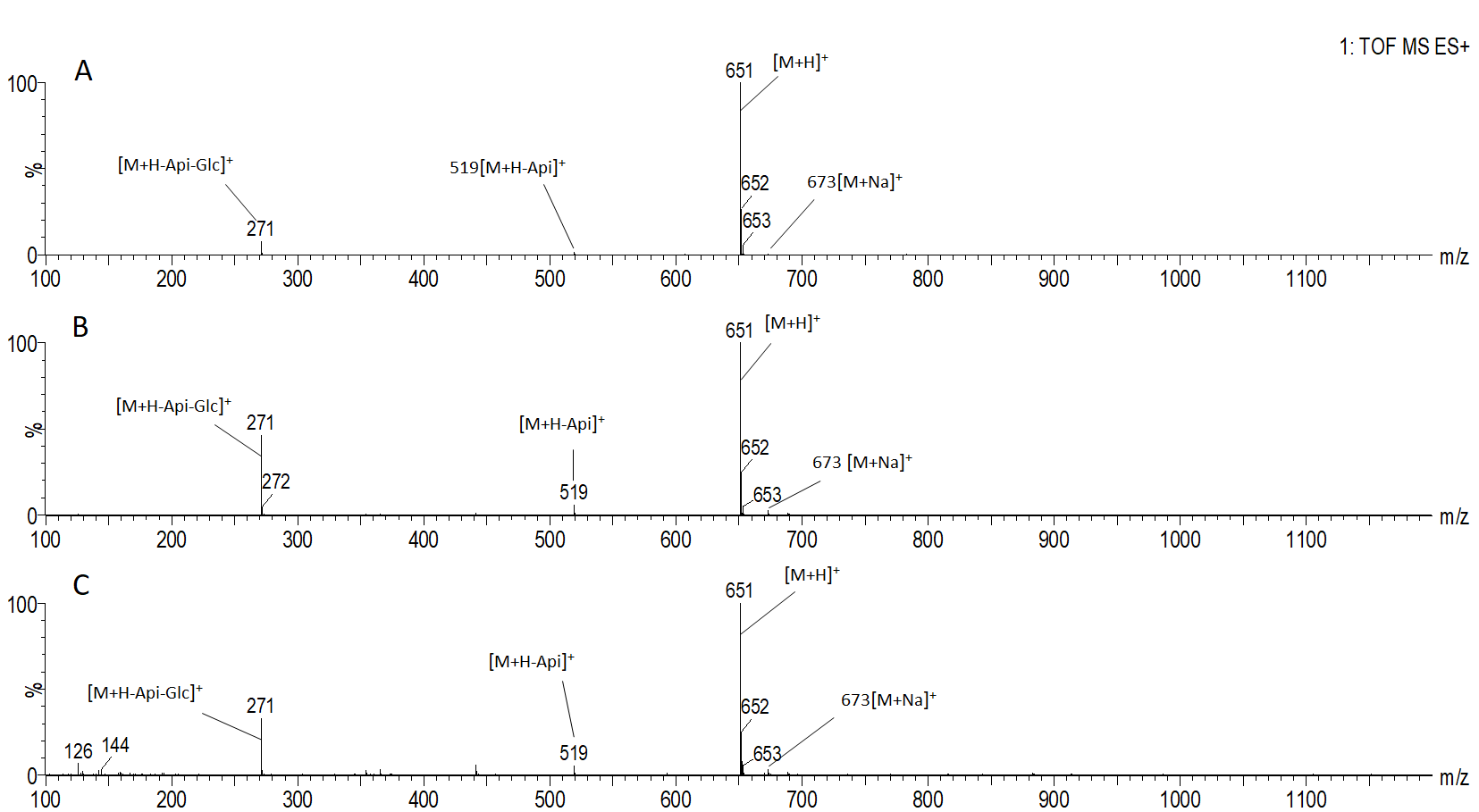

Supplement: Supplementary file 1 [file metabolites-11-00649-s001.zip › supplimentary figures/APIGENIN API MAL GLC.tif]

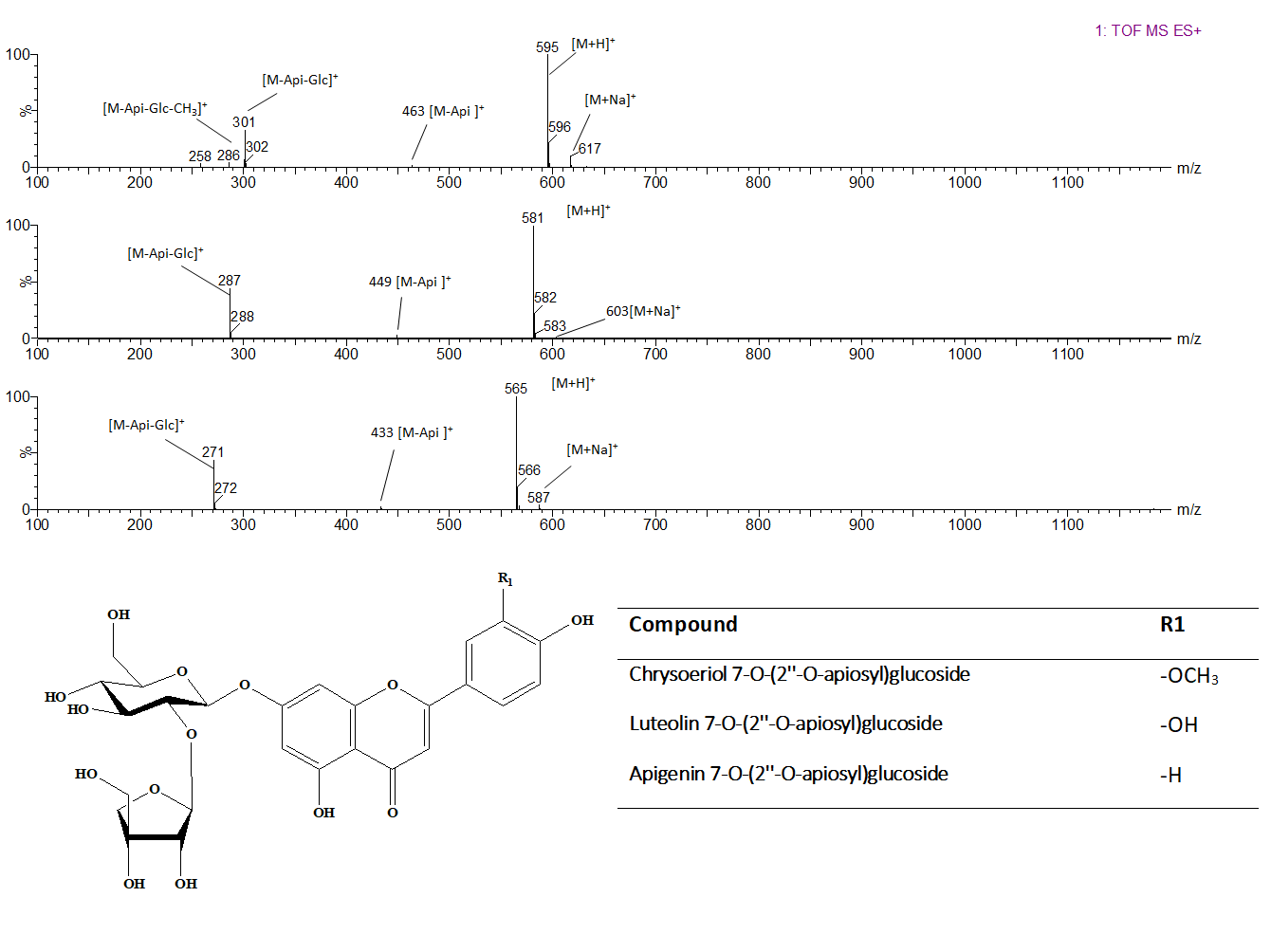

Supplement: Supplementary file 1 [file metabolites-11-00649-s001.zip › supplimentary figures/APIOSYL4.tif]

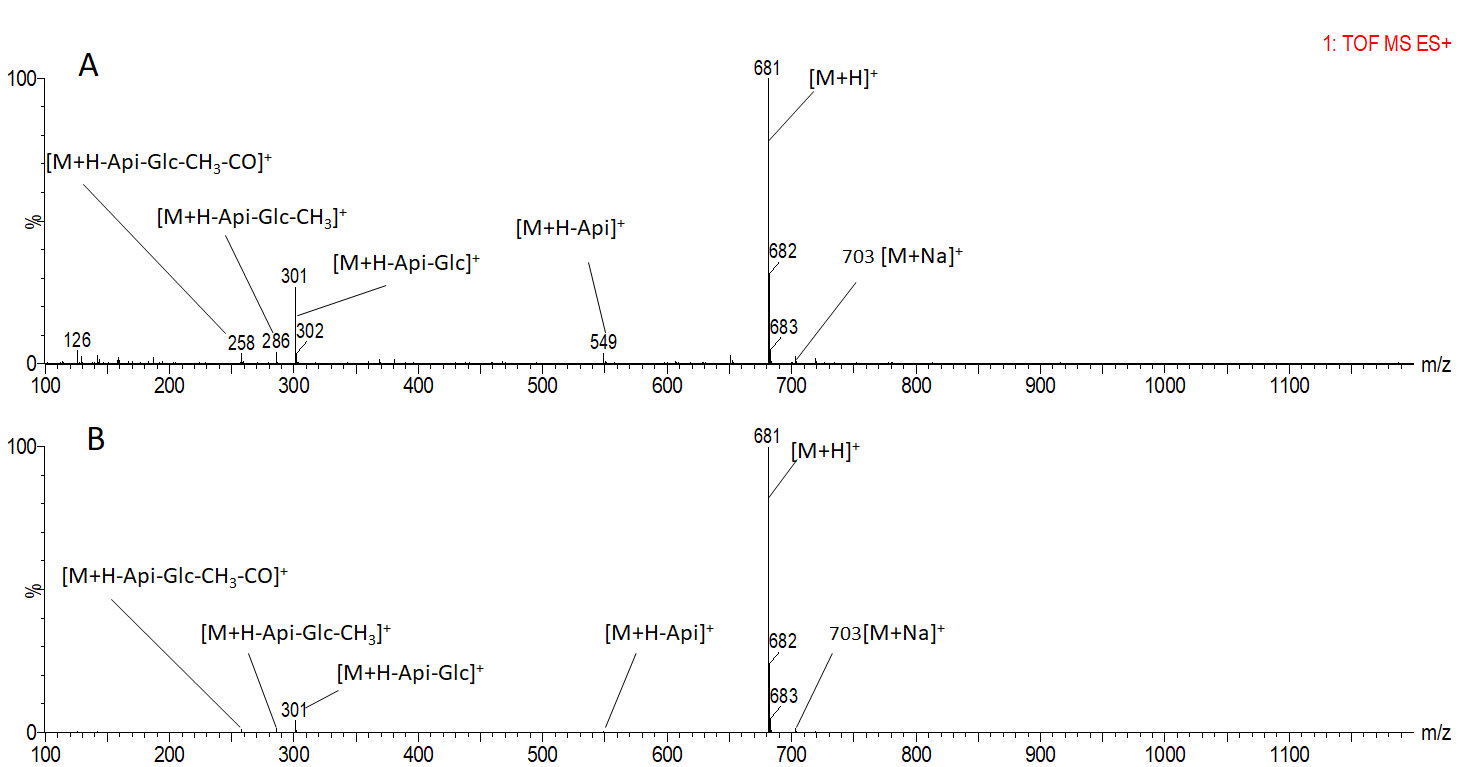

Supplement: Supplementary file 1 [file metabolites-11-00649-s001.zip › supplimentary figures/chrsoeriol isomer.tif]

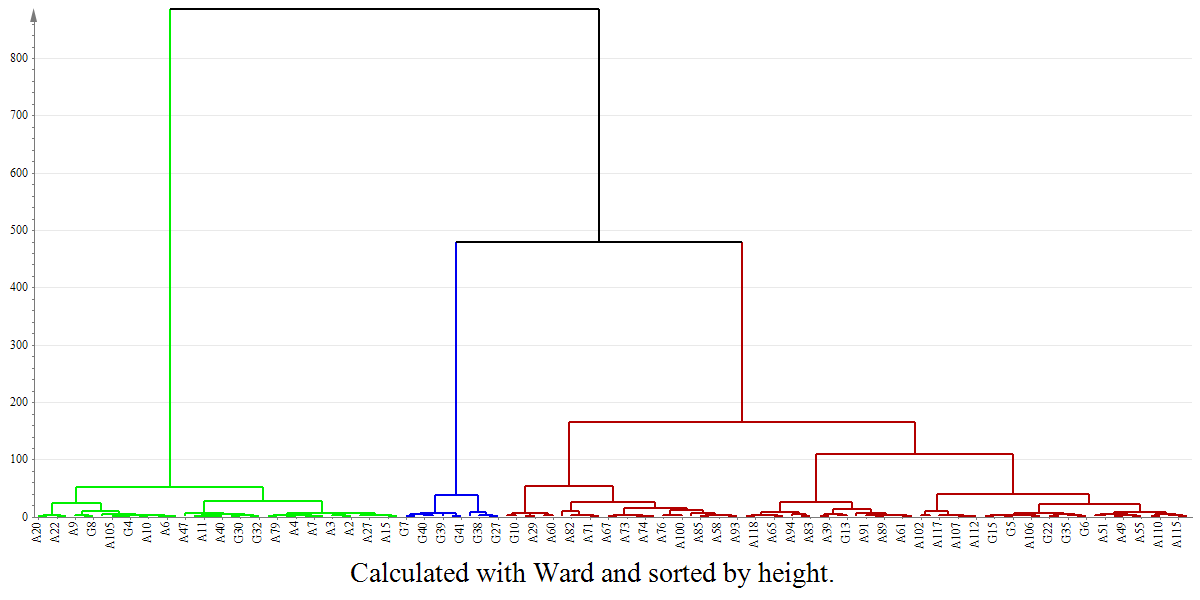

Supplement: Supplementary file 1 [file metabolites-11-00649-s001.zip › supplimentary figures/Dendrogram.tif]

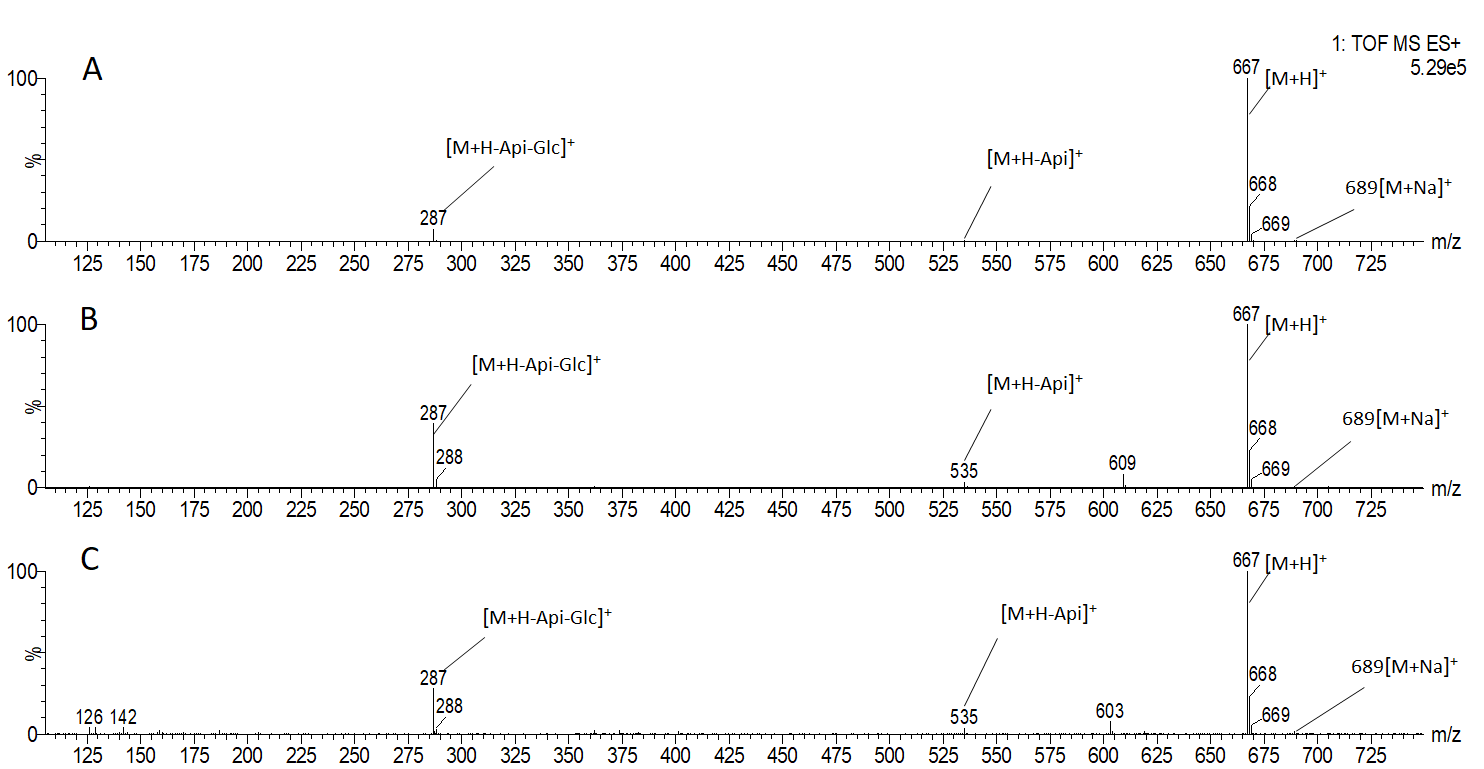

Supplement: Supplementary file 1 [file metabolites-11-00649-s001.zip › supplimentary figures/luteolin api -mal glc.tif]

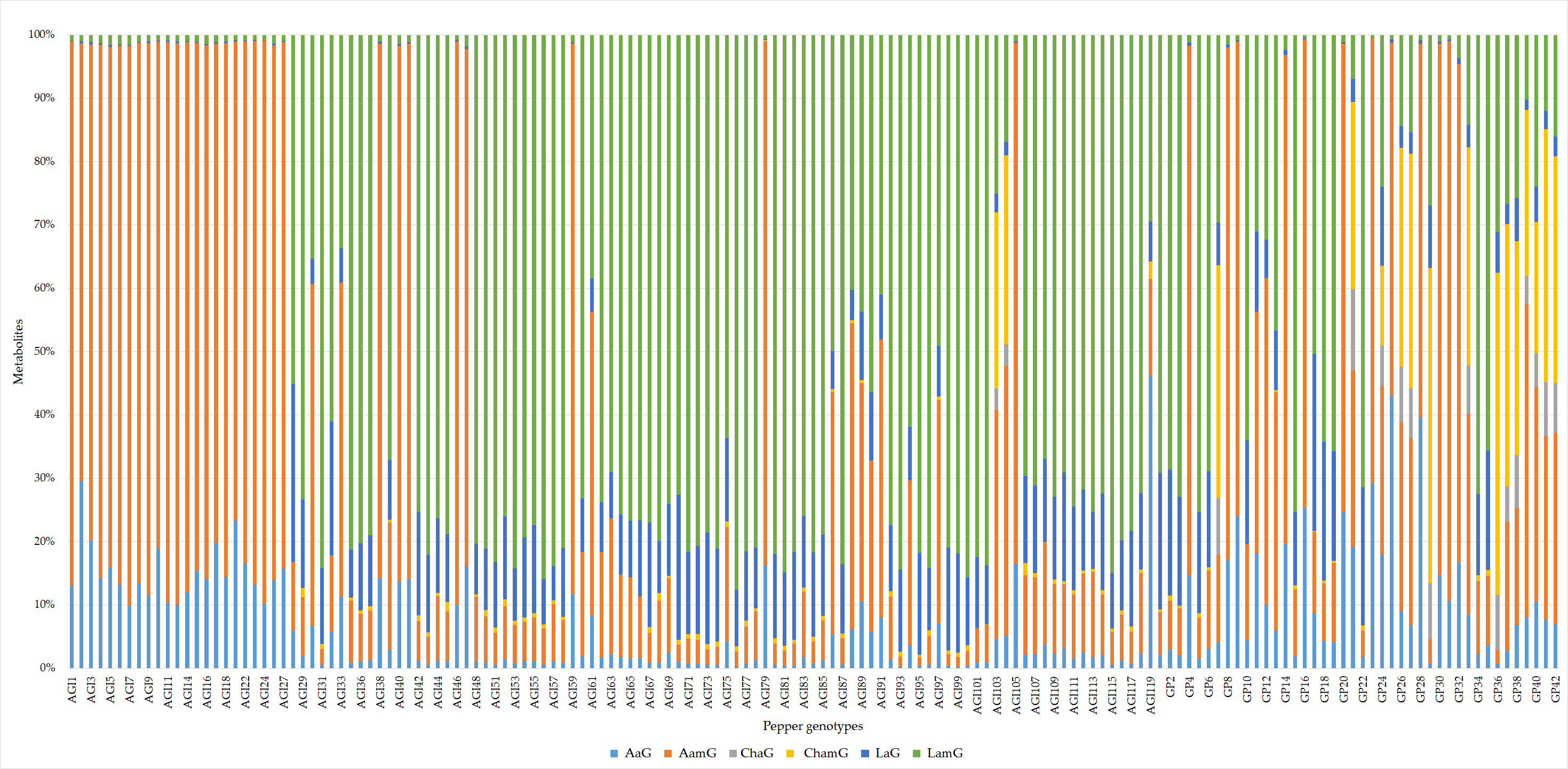

Supplement: Supplementary file 1 [file metabolites-11-00649-s001.zip › supplimentary figures/metabolite relative abundance.tif]
